# Networking between hospitals

**DOI:** 10.1007/s00068-025-02827-5

**Published:** 2025-03-26

**Authors:** Klaus Wendt, Frank Hildebrand

**Affiliations:** 1 Department of Orthopaedic and Trauma Surgery, University Medical Center Groningen, Groningen, The Netherlands; 2 Department of Orthopaedics, Trauma and Reconstructive Surgery, Frank Hildebrand, RWTH Aachen University, Aachen, Germany

**Keywords:** ESTES, Polytrauma, Whitebook

## Abstract

Not all hospitals have the resources to manage trauma patients, which is why many countries have introduced trauma systems to connect hospitals within specific regions. When definitive care cannot be provided at a hospital, the patient should be transferred to the closest appropriate facility with the necessary resources and capabilities. Telecommunication is a critical tool for data exchange (e.g., imaging) and multidisciplinary consultations. Ideally, a unified telecommunication system should be implemented across all hospitals in a network, with the long-term goal of nationwide or even European-level standardisation. While criteria for onward transfer vary slightly between trauma systems across countries, they can be adapted to meet the needs of any healthcare system. Decisions regarding patient transfer should be based on objective, prospectively agreed criteria. In addition, a common European trauma registry is essential to evaluate quality of care.

## Inter-hospital communication

Because some hospitals lack the capacity to manage severely injured patients, trauma systems linking hospitals have been developed in certain regions. Within these networks, each hospital’s facilities and capabilities are well-defined. Each trauma region should include at least one maximum-level trauma hospital equipped with the necessary resources to treat severely injured patients. If a hospital cannot provide definitive care, the patient should be transferred to the closest appropriate facility with the required capabilities. Such facilities also have an obligation to accept these patients.

Every inter-hospital transfer carries risks. Current documentation practices for inter-hospital transfers of trauma patients often lack critical physiological and ATLS variables, increasing the potential for adverse events. This underscores the need for education, training and the implementation of standardised trauma transfer protocols to improve system-wide information sharing. Robust inter-hospital communication is vital to mitigate risks and enhance outcomes.

Before a transfer, direct communication must occur between the referring and receiving surgeons. This should be conducted via phone or other communication technologies. Referring professionals should use a standardised template to provide receiving trauma surgeons with all necessary information for the patient’s further care.

Example checklist:

- WHO Acute Transfer Checklist.

In addition, referring professionals must send all available documentation (e.g., lab results, consultations) to the receiving surgeon, preferably electronically and in compliance with national data protection regulations. Diagnostic imaging already performed should accompany the patient, either via physical media (e.g., CD, DVD) or, ideally, through telecommunication. Regular training on these protocols is essential.

## Inter-hospital telecommunication (Tele-Cooperation)

Telecommunication or telemedicine supports healthcare delivery over long distances and is increasingly critical for data exchange within trauma networks. Its applications include:

- Supporting on-site treatment of severely injured patients in hospitals with limited resources.
- Facilitating the exchange of radiological data (e.g., CT scans, MRI) and enabling multidisciplinary consultations before patient transport.

In most countries, maximum-level trauma hospitals are in urban areas; thus, patients in rural regions may face longer transport times and lower chances of survival. Telecommunication bridges this gap by providing remote support and enabling hospitals with limited trauma-care capacities to offer improved initial care. This is especially vital in rural settings, where timely and accurate guidance can enhance outcomes for patient awaiting transfer to major trauma centres.

### Cross-Border Cooperation

While many countries have established national trauma networks with corresponding decreases in mortality, these networks rarely extend beyond national borders. This presents a significant challenge in Europe, where numerous borders separate neighbouring countries. Cross-border initiatives, such as the EGALURG Project (France and Spain) and Boundless Trauma Care Central Europe (Germany, Netherlands, Belgium, Luxembourg, France, and Switzerland), provide valuable insights for addressing these issues.

Key challenges include:

- Establishing cross-border trauma regions.
- Facilitating cross-border cooperation between hospitals.
- Creating a European trauma network.
- Addressing differences in laws, regulations, and financial compensation for medical care.
- Standardising quality criteria for trauma care.
- Developing a unified European trauma registry.

Collaboration between the European Union and organisations such as the European Society of Trauma and Emergency Surgery (ESTES) is critical to overcoming these challenges. Existing cross-border initiatives can serve as models for expanding European trauma networks.

### Criteria for onward transfer

Decisions to transfer patients for specialised care depend on several factors, including the nature and severity of injuries, the expected progression of these injuries, and the capabilities of the receiving facility.

#### Primary triage

The goal is to transport patients directly from the field to facilities capable of providing definitive care. However, factors including airway management, rural settings, or inclement weather may necessitate primary stabilisation at a closer facility before secondary transfer.

#### Secondary triage

Conducted at the initial receiving facility, secondary triage offers several advantages.

- It prioritises transferring the most severely injured patients to higher-level facilities, preserving resources for those in greatest need.
- It allows patients with less severe injuries to receive care closer to their own community (8).

While criteria for onward transfer differ between trauma networks, these decisions should always be based on objective, prospectively agreed criteria. Regular evaluation and training for stakeholders (prehospital care providers, hospitals) are essential to ensure consistent application of these criteria.

### Trauma registries

Comprehensive epidemiological data, treatment methods, and outcomes are essential for improving trauma care. This underscores the importance of trauma registries, which have been established in several European countries, including:

- British Trauma Audit and Research Network (TARN).
- Trauma Registry of the German Society of Trauma Surgery (DGU-TR).
- Dutch Trauma Registry (DTR).
- Norwegian Trauma Registry (NTR).

Despite progress, variations in inclusion criteria, datasets, and outcome prediction methods remain. The Utstein template for uniform reporting of major trauma data (developed in 2007) serves as a foundation, but must be built upon, to create a unified European trauma registry. Standardisation is critical for facilitating cross-border trauma care and network integration.

## Conclusion and needs for the future

Trauma systems in Europe must establish comprehensive networks where polytrauma patients are transferred to the closest appropriate hospital with the resources to provide definitive care. These networks should incorporate robust telecommunication systems to facilitate data exchange (e.g., CT scans, MRI) and multidisciplinary consultations.

Key priorities include:

- Establishing a unified telecommunication system on a national and European scale.
- Defining nationally standardised criteria for onward transfer.
- Developing a unified European trauma registry to enhance data-driven improvements in trauma care and ensure equitable outcomes across Europe.

Collaboration among European stakeholders is essential to meet these goals and address current gaps in trauma care delivery.

## Data Availability

No datasets were generated or analysed during the current study.
